# Correlated flickering of erythrocytes membrane observed with dual time resolved membrane fluctuation spectroscopy under different d-glucose concentrations

**DOI:** 10.1038/s41598-021-82018-5

**Published:** 2021-01-28

**Authors:** J. Tapia, N. Vera, Joao Aguilar, M. González, S. A. Sánchez, P. Coelho, C. Saavedra, J. Staforelli

**Affiliations:** 1 Departamento de Física, Facultad de Ciencias Físicas y Matemáticas, Universidad de Concepción, Concepción, Chile; 2 Laboratorio de Investigación Materno-Fetal (LIMaF), Departamento de Obstetricia y Ginecología, Facultad de Medicina, Universidad de Concepción, Concepción, Chile; 3 Departamento de Polímeros, Facultad de Ciencias Químicas, Universidad de Concepción, Concepción, Chile; 4 Facultad de Ingeniería y Tecnología, Universidad San Sebastián, Lientur 1457, 4080871 Concepción, Chile

**Keywords:** Membrane biophysics, Biophysics, Cell biology, Optics and photonics, Optical techniques

## Abstract

A correlated human red blood cell membrane fluctuation dependent on d-glucose concentration was found with dual time resolved membrane fluctuation spectroscopy (D-TRMFS). This new technique is a modified version of the dual optical tweezers method that has been adapted to measure the mechanical properties of red blood cells (RBCs) at distant membrane points simultaneously, enabling correlation analysis. Mechanical parameters under different d-glucose concentrations were obtained from direct membrane flickering measurements, complemented with membrane fluidity measurements using Laurdan Generalized Polarization (GP) Microscopy. Our results show an increase in the fluctuation amplitude of the lipid bilayer, and a decline in tension value, bending modulus and fluidity as d-glucose concentration increases. Metabolic mechanisms are proposed as explanations for the results.

## Introduction

Several aspects of the mechanical and micro rheological properties in red blood cells (RBCs) are still unknown. Membrane fluctuations have been understood as a random process, metabolically and thermally driven, where fluctuations in RBC membrane points are independent of each other across the lipid bilayer^1–3^. The metabolic interaction between the cell membrane and underlying spectrin mesh has been pointed to as responsible for the maintenance of the RBC biconcave shape and increased fluctuations over the thermal fluctuation below the 10 Hz regime. This process is active, dependent on the adenosine triphosphate (ATP) availability^2,4^, obtained from anaerobic glycolysis of the bloodstream dissolved glucose, which is used by the different membrane ATPases, and by the proteins that bind the membrane to the spectrin net. How these processes react to different glucose concentrations is unknown. Some conditions alter the glucose concentrations in the bloodstream, such as diabetes in all its variants, in acute or chronic form, which includes an increased risk of suffering microangiopathic disorders and macrovascular diseases^5,6^*.* The mechanical properties of the RBCs are altered by diabetes, affecting the capability of the RBCs to pass through narrow capillaries^7^. Further, humans with type 2 diabetes suffer impairment of ATP release, which is related to reduced vasodilation^7,8^. Several aspects of the effect of hyperglycemia on the mechanical and rheological properties of RBCs are unknown including the variation of the mechanical parameters and fluctuation of the RBC membrane exposed to different d-glucose concentrations, and how these alter the behavior of the entire bilipid membrane.

Owing to the participation of ATP in metabolic processes, and its availability related to d-glucose, we aim to measure mechanical properties in normal RBC as control subjects and to observe how these parameters behave in the presence of modified d-glucose concentration. In this work, we determine how the erythrocyte membrane fluctuations and mechanical parameters vary depending on the d-glucose concentration using the non-invasive d-TRMFS technique. The method is inspired by the time resolved thermal fluctuation spectroscopy (TRMFS) from Betz^2,9,10^ and the dual laser beam optical tweezers proposed by Moffitt and Bustamante^11^ for differential detection. Although d-TRMFS has the same resolution as a single TRMFS to measure the fluctuation and mechanical properties of the RBCs, our approach allows a simultaneous and non-invasively measurement on two distant points of the RBC membrane, enabling a correlation analysis on the fluctuation of both membrane points. Our d-TRMFS technique expands the possibilities of Betz et al. TRMFS because it allows the observation of the structural dynamics of the RBC beyond one single point in the membrane. By performing a frequency analysis of the signal correlations, we can explore the behavior of the whole RBC in the metabolic and non-metabolic time regimes. Significant correlations in any regime might give insight into the active and passive mechanics of RBCs. The dual measurement is achieved using two orthogonally polarized laser beams, in two diametrically separated positions of the membrane, at low optical power, without exerting optical force on the membrane, with similar resolution compared to other optical techniques^12^*,* even superior^2,9,10,13–16^*.*

Three d-glucose concentrations of 5.5, 12.5, and 25 mM were prepared to keep the experimental conditions close to the clinical concentration values. Glucose level is higher than normal for values > 6.1 mmol/L (110–125 mg/dL) and hyperglycemia for values higher than 7.0 mmol/L (126 mg/dL)^5^. Twenty independent data set of 10 s acquisition time at 20 kHz per RBC were acquired, considering that low-frequency metabolic processes occur below 10 Hz^9,10^. A model-independent correlation analysis between both signals shows an unexpected and significant correlation for RBC membrane displacement between opposite sides. These correlation values increase from high to low frequencies for each d-glucose concentration, independently. Besides, the correlation values increase comparatively between d-glucose concentrations from high to low frequencies. As the d-glucose concentration increased, we observed an increase in the fluctuation amplitude and decreased membrane tension and membrane bending modulus. These results are consistent for both diametrically separated sensing spots. Additionally, RBC membrane fluidity and volume changes were determined using generalized polarization images (GP images) and the fluorescent dye Laurdan^17^.

## Results

**Figure 1:**
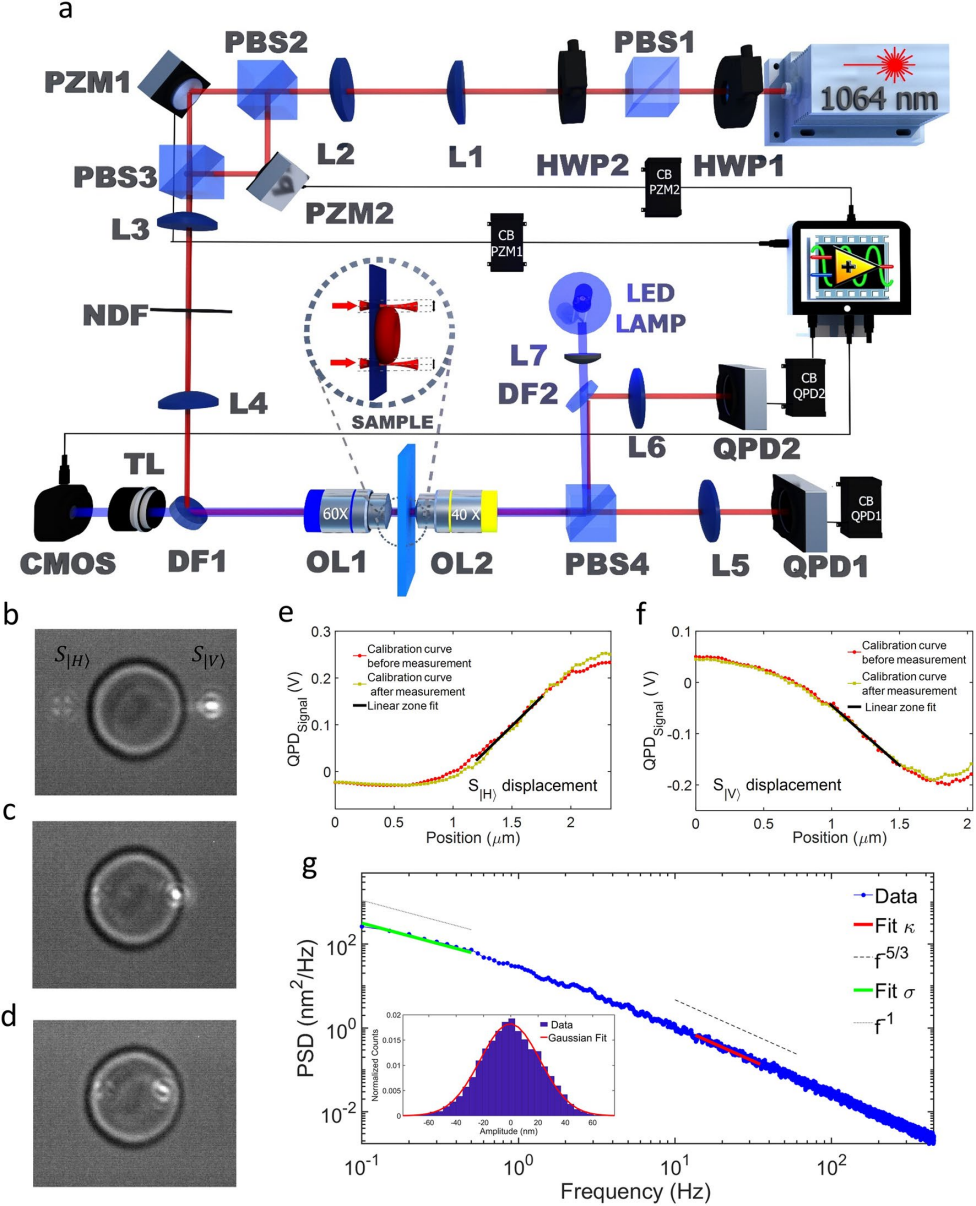
Experimental setup and method. (**a**) Instrumental setup used for fluctuation measurements. For component abbreviations, see “Materials and Methods” section (*Instrumental Setup*). (**b**, **c**, **d**) View of calibration curve acquisition for ${S}_{|H\rangle }$ and ${S}_{|V\rangle }$. As each laser spot enters the RBC, it begins to appear on each signal as a linear zone, as is shown in (**e**) and (**f**) (*Matlab ver. 2018b*). In this zone, the position changes are linearly proportional to the voltage changes in each QPD. The conversion factor Volt to nm for each spot was obtained by using the inverse of the slope (solid black line). (**g**) Example of an average power spectral density (PSD) by calculating the individual PSDs of 20 position files of 10 s each at 20 kHz of acquisition rate over a single RBC (*Matlab ver. 2018b*). Normal theoretical functions and experimental fits for low and high frequencies (under 1 Hz and over 10 Hz, respectively) are shown in accord with the plane membrane model, where low- and high-frequency zones allow the tension (σ) and bending modulus (κ) experimental values to be obtained. Theoretical functions correspond to *f*^−1^ (dotted line) and *f*^−5/3^ (dashed line), respectively, where their experimental fits have been indicated for solid lines (green and red), respectively.

Three different d-glucose concentrations were used to study the effects of d-glucose on the mechanical properties of the membrane in RBCs; 5.5 mM was considered standard control, and 12.5 mM, and 25 mM, corresponding to hyperglycemic environments. By using the d-TRMFS setup (Fig. 1a), data were obtained simultaneously in two different positions, named spots ${S}_{|V\rangle }$ and ${S}_{|H\rangle }$, which are diametrically separated in the perimeter of the RBC and have horizontal and vertical polarizations, respectively (Fig. 1b–d). The laser power is lowered to levels where optical trapping is negligible (500 μW per measurement spot). For each concentration, independent RBCs were measured in 10-s intervals at an acquisition rate of 20 kHz. The numbers of RBCs examined for 5.5, 12.5, and 25 mM d-glucose were 103, 61, and 47 cells, respectively. For further details about the experimental setup, calibration method, and models for mechanical parameters, see “Materials and Methods” section.

### Signal correlation

**Figure 2:**
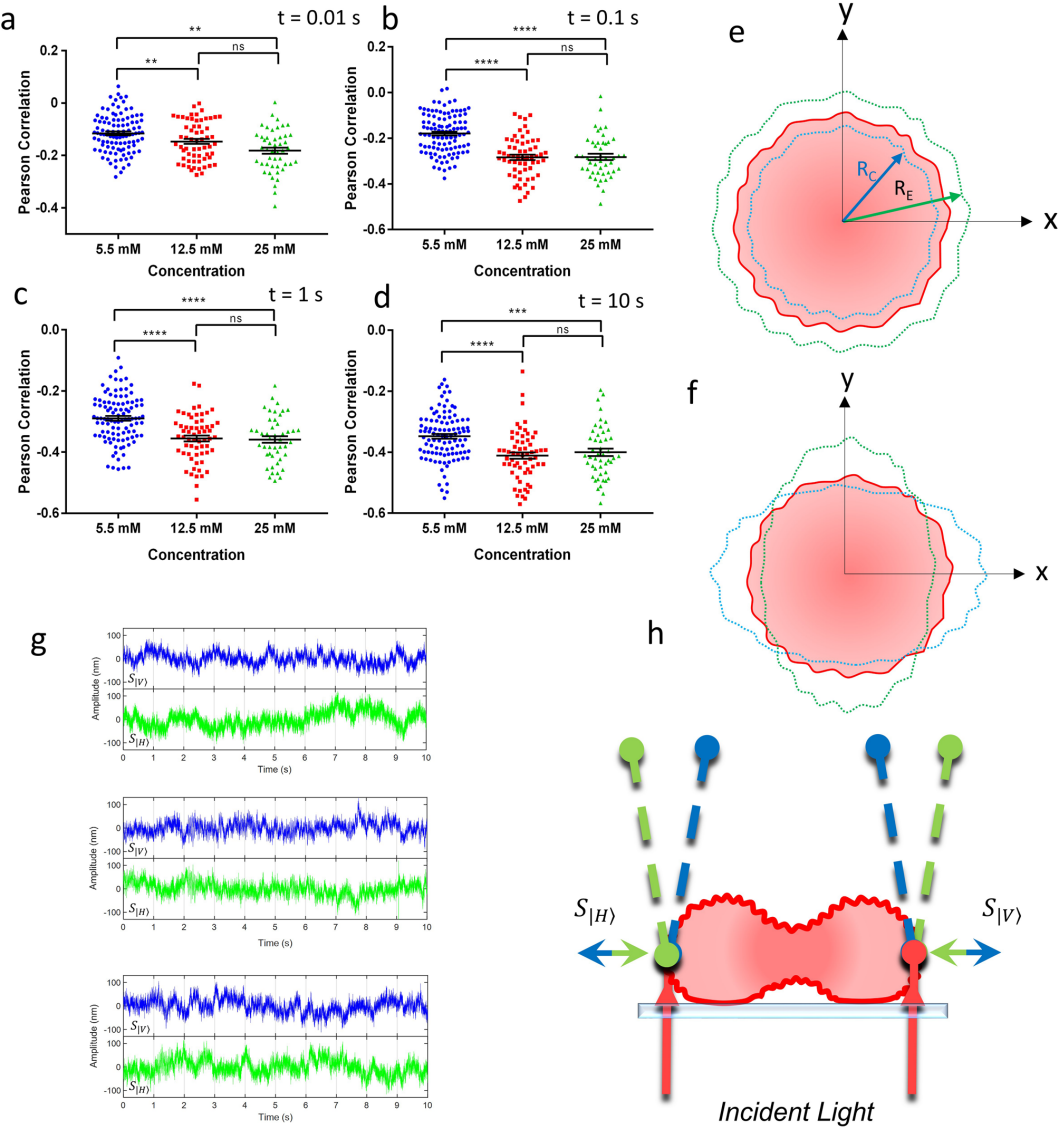
Signal Pearson’s correlation for RBCs at different D-glucose concentrations. (**a**–**d**) Correlations at different time windows associated with different low frequencies (*GraphPad Prism ver. 7*). (**e**, **f**) Two different interpretations of anticorrelated motions. (**e**) is associated with a volume change (radial expansion and contraction, ${R}_{E}$ and ${R}_{C}$, respectively), and (**f**), orthogonal vibration mode, with the dashed blue and green lines showing orthogonal deformation. (**g**) Temporal fluctuation signal amplitude ${S}_{|H\rangle }$ (green) and ${S}_{|V\rangle }$ (blue) for 5.5, 25.5, and 25 mM of glucose concentration with Pearson’s anticorrelation values of -0.38, -0.46, and -0.40, respectively (*Matlab ver. 2018b*). (**h**) Lateral view of Laser deflection. Discocyte Cell membrane beating (blue and green arrows) displaces both beam lasers in opposite directions to photodetectors. Small ripples in the membrane represent the high-frequency thermal fluctuations.

We performed a Pearson's correlation analysis for the signals as time series for spots ${S}_{|V\rangle }$ and ${S}_{|H\rangle }$ for each RBC measurement. Pearson’s correlation values varied from − 1 to + 1, where − 1 is a perfect anticorrelation, 0 is a null correlation, and + 1 is a perfect correlation. We divided the temporal signals into 0.01, 0.1, 1, and 10-s time windows to analyze different low-frequency cut at 100, 10, 1, and 0.1 Hz, respectively, for the signal correlation. Then, we obtained a correlation average for each RBC, and the results are shown in Table 1 and Fig. 2a–d. From the frequency analysis, we found that these negative correlations are predominant in the low-frequency regime, with the 10-s signal (0.1 Hz) being the lower frequency signal and 0.01second signal (100 Hz) being the highest. These anti-correlations increase from 5.5 to 12.5 and 25 mM of d-glucose concentration for all temporal windows, reaching a plateau for 12.5 and 25 mM. The anticorrelation increase for each concentration of 5.5, 12.5, and 25 mM, independently, from the high to the low frequencies with significant differences in an analysis of variance (ANOVA) test (P-values shown in *Supplementary* Table 1). Figure 2g shows a dual signal behavior for 10 s for different RBCs at 5.5, 12.5, and 25 mM d-glucose concentrations. The partial anticorrelation can be seen in reflected signal portions.

**Table 1:** Average signal correlation values for various time windows.

Time Window [s]	Concentration [mM]	Signal Pearson’s Correlation
0.01	5.5	− 0.116
	12.5	− 0.147
	25	− 0.182
0.1	5.5	− 0.179
	12.5	− 0.284
	25	− 0.282
1	5.5	− 0.289
	12.5	− 0.356
	25	− 0.356
10	5.5	− 0.348
	12.5	− 0.411
	25	− 0.400

According to the setup geometry, anticorrelated signals can be interpreted as two types of motions. The first motion approximates a radial expansion–contraction movement over time. This cell "beat" probably changes the entire cell volume. The second motion would correspond to a radial expansion–contraction movement orthogonal to the perpendicular axes. In this case, the entire cell volume can be constant. These vibration movements are depicted in Fig. 2e, f. These movements deflect the light beams in opposite directions on the quadrant photodetectors (QPDs), as shown in Fig. 2h. Even though our experiment was properly isolated from the lab floor by using an isolated optical bench, any kind of external noise that may be introduced in our experiments, such as laser wandering or foot traffic in the building, should result in a positively correlated signal due to the observed behavior of the signals in the calibration procedure and the parity reflections in d-TRMFS optical design, in contrast with the negative correlations we obtained in our measurements. A complete discussion about the different kinds of noises and their effect on the QPDs signals can be found in the *Supplementary Material*.

### Fluctuation amplitudes and mechanical parameters

**Figure 3:**
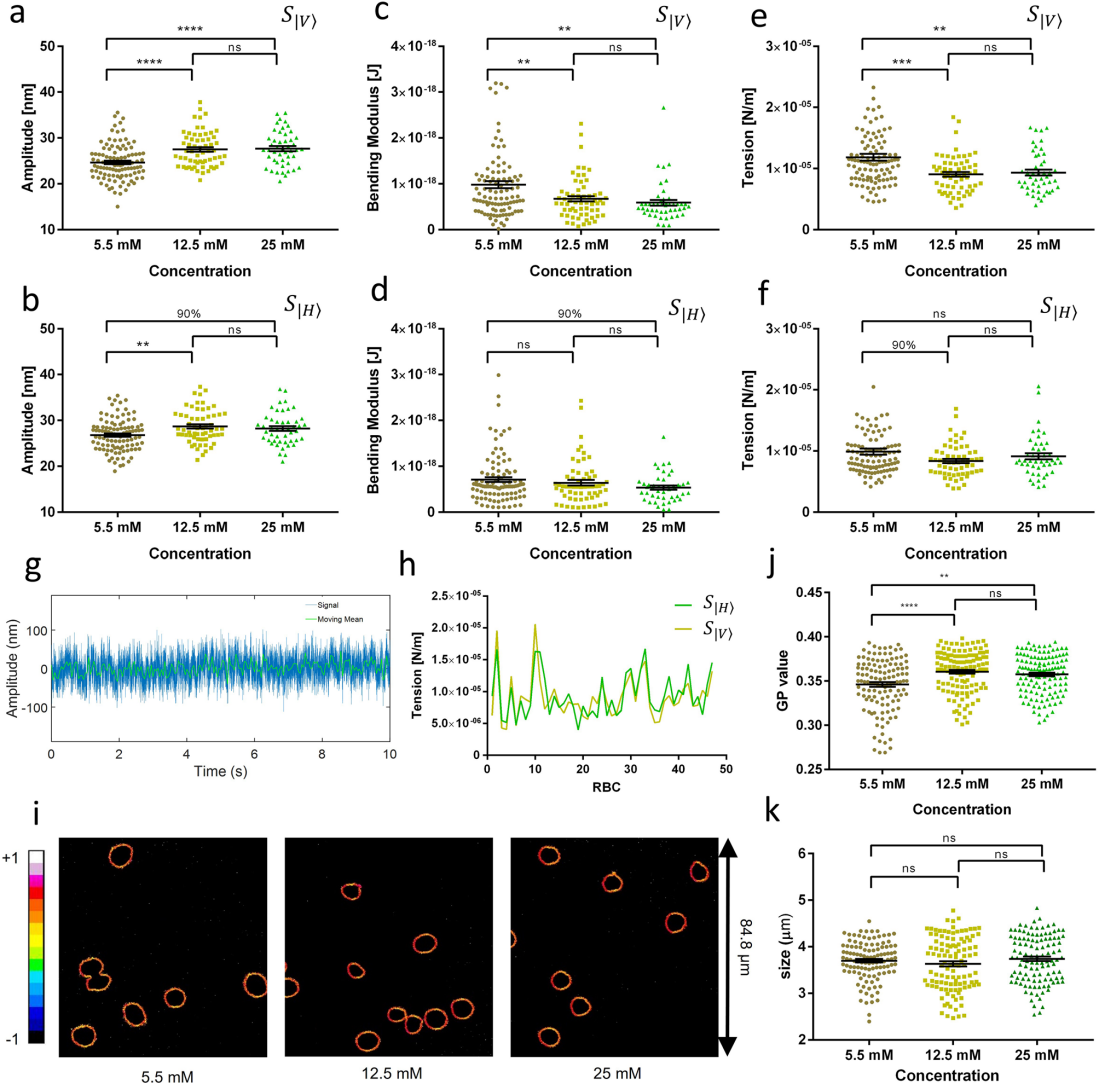
Mechanical parameters, fluidity, and size determinations for RBCs at different D-glucose concentrations. (**a**, **b**) Fluctuation amplitude distribution. (**c**, **d**) Bending modulus (κ distribution. (**e**, **f**) Tension module (σ). Data were obtained at spots ${S}_{|V\rangle }$ (**a**, **c**, **e**) and ${S}_{|H\rangle }$ (**b**, **d,**, **f**). Three concentrations of D-glucose were used: 5.5 mM (brown), 12.5 mM (yellow) and 25 mM (green). (**g**) A representative example of an amplitude signal versus time in seconds (*Matlab ver. 2018b*). The moving mean is highlighted in green, and the moving standard deviation is a dashed black curve. (**h**) Simultaneous tension values for both spots ${S}_{|V\rangle }$ (mustard) and ${S}_{|H\rangle }$ (green) obtained in individual RBCs. (**i**) Representative Laurdan GP images for the three D-glucose concentrations used (*ImageJ ver. 1.46o*). An arbitrary color scale is used for the GP values ranging between − 1 and + 1. (**j**) Fluidity (GP values) for individual RBC versus D-glucose concentration (N = 125 for each treatment). (**k**) Erythrocyte size for individual RBCs (N = 112 for each treatment) for the three D-glucose treatments. For the ANOVA test, the following parameters were used for P-value confidences. ‘**’ P = 0.001 up to 0.01, or 99%; ‘****’ is P < 0.0001, > 99.9%; and ns means ‘no significance’. The calculations of P-values, mean values and mechanical parameters were performed with these non-artifact data, which are RAW data. Symbols: from the ANOVA test (*GraphPad Prism ver. 7*), we used P-value confidences. 90% corresponds to P-values between 0.05 and 0.1; ‘*’ is P = 0.01 up to 0.05 of confidence, or 95%; ‘**’ is P = 0.001 up to 0.01, or 99%; ‘***’ is P = 0.0001 up to 0.001 or 99.9%; ‘****’ is P < 0.0001, > 99.9%; and ns means ‘no significance’.

Following the protocol described in “Materials and Methods” section and according to Eqns. 1 and 2 from the membrane model considered in this work^2,9,10^, Table 2, and Fig. 3 summarize mean fluctuations and the membrane mechanical parameter obtained for RBCs at different d-glucose concentrations. For the control (5.5 mM), the mean fluctuation values were 24.66 $\pm$ 0.38 nm and 26.81 $\pm$ 0.35 for spots ${S}_{|V\rangle }$ and ${S}_{|H\rangle }$, respectively. From 5.5 mM to 12.5 mM, the fluctuation amplitudes grew 11.5% and 7% for ${S}_{|V\rangle}$, and ${S}_{|H\rangle }$, respectively. From 12.5 mM to 25 mM, there was no significant difference for ${S}_{|V\rangle }$ or ${S}_{|H\rangle }$ (Fig. 3a, b). The bending modulus (Table 2 and Fig. 3c, d) decreased 31% for ${S}_{|V\rangle }$ and 9.5% for ${S}_{|H\rangle }$ from 5.5 mM to 12.5 mM. From 12.5 mM to 25 mM d-glucose, the bending modulus showed no significant difference for either ${S}_{|V\rangle }$ or ${S}_{|H\rangle }$. From 5.5 mM to 25 mM, the bending modulus showed the most significant difference for both ${S}_{|V\rangle }$ and ${S}_{|H\rangle }$. The tension values (Table 2 and Fig. 3e, f) decreased approximately 24% and 15.5% for ${S}_{|V\rangle }$ and ${S}_{|H\rangle }$, respectively when the d-glucose concentration changed from 5.5 mM to 12.5 mM. From 12.5 mM to 25 mM, no significant changes were observed for either ${S}_{|V\rangle }$ or ${S}_{|H\rangle }$. From 5.5 mM to 25 mM, this parameter showed a significant difference only for ${S}_{|V\rangle }$. For ${S}_{|H\rangle }$, the tension value recovered partially to a value similar to that at 5.5 mM. *Supplementary* Table 2 summarizes the corresponding P-value confidences for the fluctuation amplitude, bending modulus ($\kappa$), and tension parameter ($\sigma$) between the concentrations.

**Table 2:** Membrane mechanical parameters. Comparison between the average mechanical parameters for spots ${S}_{|V\rangle }$ and ${S}_{|H\rangle }$ at three different D-glucose concentrations.

Signal	Parameter	5.5 mM	12.5 mM	25 mM
${S}_{|V\rangle }$	Amplitude (nm)	24.66 ± 0.38	27.50 ± 0.49	27.68 ± 0.55
	κ (1E−19 J)	9.79 ± 0.78	6.75 ± 0.59	5.87 ± 0.63
	σ (1E−06 N/m)	11.85 ± 0.55	9.05 ± 0.39	9.35 ± 0.50
${S}_{|H\rangle }$	Amplitude (nm)	26.81 ± 0.35	28.69 ± 0.46	28.25 ± 0.52
	κ (1E−19 J)	7.07 ± 0.52	6.40 ± 0.61	5.33 ± 0.44
	σ (1E−06 N/m)	9.89 ± 0.51	8.36 ± 0.39	9.11 ± 0.50

Mean values are presented with the standard error of the mean (SEM).

We calculated Pearson’s correlation for each average membrane parameter for both signals ${S}_{|V\rangle }$ and ${S}_{|H\rangle }$, the values are shown in Table 3. We obtained moderate to high degree correlation values for the different parameters at different d-glucose concentrations. For academic purposes, tension values per RBC number at 25 mM for both signals are shown in Fig. 3h. This graphic and the values in Table 3 confirm that the RBC membrane has from moderate to high degree of average mechanical homogeneity around the rim. It is essential to clarify that these results correspond to an average effect, which is not in conflict with the existence of inhomogeneities in the lipid bilayer, such as microdomains and membrane sub-structures.

**Table 3:** Pearson’s correlations for mean mechanical parameters.

Parameter	Amplitude			Bending mod. (κ)			Tension ($\sigma$)		
Concentration [mM]	5.5	12.5	25	5.5	12.5	25	5.5	12.5	25
Pearson’s Correlation	0.433	0.456	0.599	0.625	0.474	0.358	0.795	0.526	0.802

### Membrane fluidity determined by Laurdan generalized polarization

The TRMFS technique does not evaluate the intra-membrane fluidity. Complementing the results about d-glucose's effect, membrane fluidity was determined using generalized polarization images (GP images) and the fluorescent dye Laurdan^17^. Dead RBC or echinocytes were not considered; only discoid cells were analyzed (similar to the fluctuation analysis). In the image analysis, only a ring of 5 pixels taken from the outside rim to the center of the RBCs was considered (Fig. 3i) to minimize the cytoplasm's contribution in the analysis. Average GP values for each treatment are shown in Fig. 3j. In addition to the dispersion of the data, the average GP values (center of the histogram distribution) for each treatment are shown. A small but statistically significant increase in the average GP value was observed for d-glucose 12.5 mM compared with 5.5 mM. A further increase to 25 mM d-glucose did not change the average GP value, reaching a plateau between 12.5 and 25 mM d-glucose. An increase (decrease) in the GP value corresponds to a decrease (increase) in the fluidity value.

Possible changes in the size of the RBCs due to the incubation with d-glucose were analyzed. Individual RBCs from the intensity images (N = 112 for each treatment) were used to calculate the sizes of the RBCs (Fig. 3k). No significant changes in radius were observed between the three treatments, and the values for RBC radius were (3.69−/ + 0.04), (3.63 + /- 0.05), and (3.74 ± 0.05) µm for incubation with 5, 12.5, and 25 mM d-glucose, respectively. It is essential to mention that confocal images for size analysis were obtained in one z-plane (at the center of the RBCs); therefore, slight changes in volume in the axial direction might be undetectable. The resolution in a two-photon microscope is given by the point spread function (PSF) of the illumination beam and is around 400 nm in diameter (XY plane) and 2–3 times this value for Z-axis.

## Discussion

The increasing anticorrelations for the > 0.1 s time windows in Table 1 coincide with the range of out of equilibrium metabolic processes in RBC while the 0.01 s time window should coincide with thermal fluctuations. This result suggests that the anticorrelations have a metabolic, non-thermal origin. Also, the anticorrelations are stronger for the higher d-glucose concentrations. This fact supports that these coordinated low-frequency movements could be triggered by non-equilibrium activities that modulate the entire cell. For the 0.01 s time window (100 Hz), there is no significant correlation as expected for thermal processes. It is necessary to remark that these correlation results are model-independent. Accordingly, with the correlation results, we obtained larger membrane fluctuation amplitudes for increased d-glucose concentrations (Fig. 3a, b), and both show a saturation plateau between 12.5 and 25 mM. The metabolic activity has been understood as a metabolic noise, with stochastic properties. Here we see that the increasing anticorrelations suggest a non-stochastic process in spectrin net remodeling. Concerning fluctuation amplitude, it has been related to the metabolic remodeling of the spectrin network through the phosphorylation of the 4.1 protein and ankyrin by the increased availability of ATP by anaerobic glycolysis^4,18,19^*.* This activity models the RBC in the discocyte shape, as shown in Fig. 2h. Activities of **Na+/K+** and **Ca**^**2+**^ ATPases membrane proteins, among others, are also related to the increased fluctuation^1,20^. On the other hand, the studied allosteric processes guarantee a tight control of glycolysis^21,22^. It is reported that RBCs energy consumption occurs at a constant rate, independent of the external glucose concentration. The allosteric mechanism could be affected by the differences in glucose concentrations, resulting in increased energy consumption. Regarding the observed plateau effect, we see an active processes increment in the 12.5 mM d-glucose concentration and saturation at 25 mM, which may be showing saturation due to the allosteric mechanism.

Although the ATP concentration, as a function of the d-glucose concentration, was not measured in this work, it is possible to interpret that higher d-glucose concentrations would allow greater ATP availability after 24 h of incubation. Therefore, this analysis is in accord with our results.

According to Table 2 and Fig. 3c, d, membrane bending modulus decreases as d-glucose increases. Previous works related the bending modulus behavior to ATP availability^2,4,23–25^, however, it is a passive membrane parameter fitted in the high frequencies regime (> 10 Hz, see “Materials and Methods” section) and its dependence is not directly linked to active processes. It has been proposed that the active spectrin net remodeling partially decouples the membrane and cytoskeleton rigidity, resulting in a lower measured rigidity for the membrane.

According to Table 2 and Fig. 3e, f, the membrane tension parameter decreases as d-glucose increases. On the other hand, the Laurdan results show a decrease in the intra-membrane fluidity. Membrane tension and fluidity have been related before to the membrane anisotropy^26^ and a decreasing of the fluidity has been observed when RBCs are incubated in high glucose concentration^27^. When the lipid bilayer is stretched, there is an increased space between phospholipids, which allows a higher membrane fluidity^28^. In our case, the results agree with references^26–28^, as the tension decreases jointly with fluidity for higher glucose concentrations.

It is worth note that for 12.5 mM, both parameters reach a minimum, which stays similar for 25 mM. The plateau effect is also seen across the tension and fluidity parameters. This result shows a correspondence with the work of Watala, C.^21^, where this plateau is seen in fluidity and glycation measurements as a function of glucose concentration. Glycation is a nonenzymatic reaction in which free amino groups of proteins, lipids, and nucleotides are modified by monosaccharides, like d-glucose. This reaction modifies and alters the function of proteins and lipids of membranes, leading to the formation of advanced glycation en d-products (AGEs), and it is implied in the development of pathological conditions, especially in diabetes and aging^29^. The formation of AGEs results from different pathways which included oxidative stress and lipid peroxidation, and AGEs itself contributes to increased oxidative stress^30^, especially in patients with diabetes mellitus^31^. In an animal model of diabetes, it has been determined that the fluidity (determined by 1,6-diphenyl hexatriene (DPH) fluorescent probe) of intestinal brush border membrane was significantly lower in diabetic animals compared with controls, associated with higher lipid peroxidation and protein oxidation^32^. Also, a decrease of membrane fluidity was determined in sarcolemmal membranes isolated from hearts of diabetic rats, associated with oxidative stress and glycoxidation in heart tissues^33^. In RBC from patients diagnosed with coronary artery disease, a decrease has been determined in the plasma membrane fluidity, compared with healthy controls, which were associated with higher peroxidation of lipids and lower activity of antioxidant enzymes^34^. Concerning oxidative stress, it is well known that 25 mM d-glucose induces the activity of oxidant enzymes in vascular cells, mainly related to higher production of superoxide and peroxynitrite^35^. So, oxidative stress and lipid peroxidation could be a relevant mechanism underlying the changes in plasma membrane fluidity induced by hyperglycemia. Further studies are necessary to determine if these molecular mechanisms are involved in the decrease of bending modulus and plasma membrane fluidity reported in this study.

A possible origin for the decrease of tension as glucose increases is related to cytoskeleton detachment, affecting the tension in the same way as it does with the bending modulus. The RBC membrane-cytoskeleton cohesion depends on molecular interaction between β-spectrin and band 3-ankyrin^36^. Band 3 is a plasma membrane integral protein and its interaction with ankyrin is decreased with lower oxygen availability^37^. Interestingly, the expression of band 3 is decreased in RBCs from diabetic patients and the incubation of RBC with 15 or 35 mM d-glucose decreased the activity of band 3 associated with oxidative stress and hemoglobin glycation^38^. Also, it has been reported that β-spectrin, band 3, and ankyrin are proteins highly susceptible to post-translational modifications associated with oxidative stress^39^ and the distribution of spectrin in the RBC membrane in diabetic patients^40^. Together, this evidence shows a link between oxidative stress and glycation in hyperglycemia with mechanical alterations of the RBC membrane related to altered function and interaction of β-spectrin-band 3-ankyrin complexes.

Despite that the Laurdan image analysis shows that increasing d-glucose did not produce radius changes in the general RBC population, other possible explanations might be in the osmotic balance, which is altered by the imbalance between inner and outer glucose concentrations. Here, considerations about the model used should be taken into account. At low frequencies, a practical consequence of using the passive membrane model is that the general tension parameter fitting might be underestimated because of the low-frequency metabolic activity^1,2^. The tension parameter fits for low frequencies, and the lowering tension with higher glucose concentration might be taken into account for increased fluctuations.

On differences in statistics from measurement spots and polarization, there is a notorious difference in the statistical distribution for the mechanical parameters between the two spots. In general, the results from ${S}_{|V\rangle }$ have better statistical separations between results, as seen from the ANOVA tests (Table S2); for example, the mean fluctuation amplitude is more significant for ${S}_{|H\rangle }$, and the mechanical parameters are smaller than ${S}_{|V\rangle }$ . The only asymmetry present in the experimental setup is the polarization for both spots. This may be due to the polarizability of the membrane in the presence of water and solutes from the phosphate-buffered saline (PBS) solution. The orientation of light polarization with respect to the membrane may be altering the mechanical properties. The ${S}_{|H\rangle }$ polarization is perpendicular to the cell membrane, so it may be polarizing the interaction between the membrane and the media, diminishing the effective viscosity between the membrane and the surrounding medium, resulting in a higher fluctuation amplitude. Notice that this mechanical alteration occurs at low laser power. In the context of optical tweezers, it is known that the polarization of light alters the trapping potential symmetry in critical mixtures^41^. It is important to notice that, even if this is a disadvantage for the detection system, it shows a possible effect of light polarization on membranes. Also, the correlations' values are not affected by this phenomenon due to the invariance of the Pearson correlation under scale changes.

There is an ample investigation into the effects of biological molecules on light polarization; conversely, the effects of light polarization in biological structures have not been well studied. From a physical point of view, it will be relevant to study the effects of light polarization orientation on the membrane mechanical parameters. A simple experiment using the same setup for d-TRMFS can be performed to confirm these effects. In this new experiment, an interchange of both polarized beams can be realized to account for possible mechanical differences.

## Final remarks

Our d-TRMFS implementation allowed us to observe the structural dynamic of the RBCs under the different d-glucose concentrations, expanding the possibilities of the single point TRMFS by Betz et al. The results suggest that the observed correlations are related to the RBCs non-equilibrium fluctuations.

Complementary experiments can be implemented in the same d-TRMFS setup to gain further insight into RBCs behavior. The dynamics in an ATP depleted RBC using our d-TRMFS would allow studying it in a non-energy condition. In this way, the measured signals corresponding to the membrane fluctuations in two diametrically separated places would have an exclusively thermal nature. The Pearson correlation of these signals of stochastic nature should show a non-significant correlation. Also, the light polarization effects on the membrane can be explored with a slightly modified version of the d-TRMFS, allowing the exploration of the interaction between electric fields and the bilipid membranes. This would consist of modifying the polarization of both beams before it arrives at the sample and recovering it back to the detection stage.

A further multiplexation of measurement points, as is common in the optical tweezers context, would allow the observation of the vibrational modes of the RBC, as proposed by Turlier et al.^1^.

An advantage we notice about our implementation is that we can explore the non-equilibrium dynamics in a relatively fast fashion, considering other model-independent implementations^1^. This could be used in other contexts where it is necessary to study large RBCs populations, for example, in the study of RBC aging in blood banking.

## Materials and methods

### Time resolve membrane fluctuation spectroscopy (TRMFS)

All experiments in this work conform to the principles outlined in the Declaration of Helsinki and were approved by the Ethics, Bioethics, and Biosecurity Committee of the University of Concepción.

#### Sample preparation

Adult RBCs were obtained from a healthy donor volunteer after signing informed consent. RBCs were incubated for 24 h at 37 °C in three different d-glucose (Sigma-Aldrich, St. Louis, MO, USA) concentrations, 5.5 mM, 12.5 mM, and 25 mM. This concentration range is based on physiological and pathophysiological glycemia^5,42^. Between 3.9 and 5.5 mM is considered normal. These concentrations are the conversion of range 70–100 mg/dL that is considered normal for fasting glucose in clinics. The concentration of 12.5 mM (225 mg/dL) is in the range of hyperglycemia after meals (in a diabetic patient). This range is between 11 and 15 mM or 200 and 270 mg/dL. The higher concentration of D-glucose (25 mM of 450 mg/dL) is related to dangerous complications of diabetic patients that could involve a medical emergency for the patient. A sealed chamber consisting of two coverslips was fabricated to contain the incubated RBC solution using 18 × 18 mm^2^ (top) and 24 × 50 mm^2^ (bottom) coverslips from Hirschmann Laborgeräte GmbH & Co. KG (Eberstadt, Germany). In brief, the protocol used was the following: 10 μL of total blood was diluted 100 times by adding 990 $\mu L$ of phosphate-buffered saline (PBS) solution [mM: 130 **NaCl**, 2.7 **KCl**, 0.8 **Na**_**2**_**HPO**_**4**_, 1.4 **KH**_**2**_**PO**_**4**_, **pH** 7.4 (Sigma-Aldrich)]. For incubation (37 °C, 5% **CO**_**2**_/95% **O**_**2**_, 24 h), a second dilution was prepared with 50 μL of 100-times diluted blood plus 950 μL of PBS (final dilution 1/2000) and d-glucose to final concentrations of 5.5 mM, 12.5 mM or 25 mM d-glucose, respectively (d-glucose stock of 1 M, prepared in PBS). Each sample preparation was left to decant for at least 30 min onto the bottom coverslip of the chamber.

#### Instrumental setup

The instrumental setup is shown in Fig. 1a. A Ventus HP laser (from Laser Quantum) centered at λ = 1064 nm goes through a λ/2–PBS1-λ/2 (half-wave plate (retarded) – polarized beam splitter 1—half-wave plate) system to control incident beam power to reach the low power necessary for the TRMFS and to achieve beam power balance (intensity symmetry). The beam is expanded by a telescopic system composed by lenses L1 and L2, and then split by the polarized beam splitter PBS2. Each PBS2 output is reflected by a piezoelectric mirror (PZM) for beam position control at the sample. Both beams are recombined at polarized beam splitter PBS3 and aligned to the objective lens OL1 back focal entrance (UPlanSApo, 60X/1.2 WI, Olympus, Tokyo, Japan), and the laser spot displacement is optimized on the sample plane (laser beam waist diameter with a radius of approximately 500 nm). The telescopic system is composed of lenses L3 and L4 and allows them to conjugate the PZMs plane to the OL1 back focal plane. The dichroic mirror D1 reflects the laser beam and transmits the illumination lamp IL to camera C. At the sample, the tightly focused beams pass tangentially to the membrane border of the cell. The objective lens OL2 (40X/0.65 Nikon, Tokyo, Japan) collects both beams, which are projected separately to the quadrant photodetectors QPD1 (for ${S}_{|H\rangle }$) and QPD2 (for ${S}_{|V\rangle }$) by the polarized beam splitter PBS4. Labview Software from National Instruments was used for automation and control. This scheme is an improved TRMFS setup used previously in an RBC study^43^, and it is inspired by the differential detection of dual traps scheme by Moffitt et al*.*^11^*.*

#### Calibration procedure

For each RBC, the linear working zone was determined twice, at the beginning and end of the measure, to confirm that the same linear zone was maintained. Finding the linear zone (Fig. 1e, f), a voltage sweep from 0 to 150 V was applied to each PZM (Fig. 1a). The sweep was divided into 2 V, 1-s duration steps at a 20 kHz acquisition rate, giving a 75-s measurement for the calibration curve. This sweep corresponds to 2.03 µm and 2.33 µm for spots ${S}_{|V\rangle }$ and ${S}_{|H\rangle }$ , respectively, as shown in Fig. 1b–d.

#### Membrane model

Our membrane model is based on the works of Betz et al.^2,9,10^. The fluctuation amplitude power spectral density (PSD) function for a plane membrane is

$$PSD = \frac{{4\eta {K_B}T}}{\pi }\int\limits_{{q_{\min }}}^{{q_{\max }}} {\frac{dq}{{{{\left( {\kappa {q^3} + \sigma q} \right)}^2} + {{\left( {4\eta \omega } \right)}^2}}}} ,$$

where ${K_B}$ is the Boltzmann constant, *T* is the effective temperature, κ and σ are bending modulus and tension parameters, fluctuation *q*-modes have a relaxation frequency $\omega = 2\pi f$_,_ which corresponds to an impermeable membrane, and η is the effective viscosity between the medium and the membrane. From this expression, two approximations appear, for high frequencies (ω → ∞),

1 $$PSD = \frac{{{K_B}T}}{{6\pi {{\left( {2{\eta^2}\kappa } \right)}^{1/3}}{\omega^{5/3}}}},$$.

and low frequencies (ω → 0),

2 $$PSD = \frac{{{K_B}T}}{2\sigma \omega }.$$

#### Data processing

For each RBC, 20 data files of 10 s at 20 kHz of acquisition rate were obtained. The average value of the standard deviation (STD) of all files was calculated. This value corresponds to the average fluctuation of the membrane around its equilibrium position. This average was performed for each RBC. The final STD amplitude was calculated by averaging the values obtained from all RBC measurements at each concentration.

To determine the bending modulus and tension value at a given concentration, a PSD analysis was performed by using the fast Fourier transform (FFT). For each RBC, an average PSD of all files was calculated and fitted by using the model equations for high frequencies (Eq. 1) and low frequencies (Eq. 2), which are fit regimes associated with $\kappa$ (bending modulus) and $\sigma$ (tension value), respectively. Fit results are shown in Fig. 1g. The final bending modulus and tension values were calculated by averaging the values obtained from all RBCs at each concentration.

#### Statistical analysis

The results of amplitudes and mechanical parameters are presented by using the mean value and its standard error (SEM). For each concentration, an independent number of RBCs was measured. A concentration of 5.5 mM was considered as the control. Initially, a quantity of N = 114 RBCs was measured for the 5.5 mM concentration, with quantities of N = 65 and N = 49 for 12.5 mM and 25 mM, respectively. A procedure to detect outliers was applied on mean fluctuation amplitude values for each concentration using a standard quartile method, which consists of using the interquartile range. A mean value is considered an outlier when it is out of an upper or lower limit defined as the third quartile plus 1.5 times the interquartile range and the first quartile minus 1.5 times the interquartile range, respectively. For the control at a concentration of 5.5 mM, N = 103 RBCs were considered for fluctuation and tension analyses, and N = 102 RBCs were considered for bending modulus analysis. For d-glucose concentrations of 12.5 mM, and 25 mM, N = 61 and N = 47 were considered, respectively. Data were then analyzed by one-way ANOVA, and a comparison between groups was performed using a protected Tukey test. The data treatment carried out in this work is similar to that carried out by Betz^2^ and other similar authors, where results are also presented with SEM^44^.

### Laurdan generalized polarization imaging

Laurdan 6-lauroyl,1–2-dimethylaminonaphthalene is a fluorescent molecule used to detect changes in membrane phase properties through its sensitivity to the intra-membrane polarity^45^. Polarity changes are shown by shifts in the Laurdan emission spectrum, which are quantified by calculating the generalized polarization (GP) Eq. ^45–48^.

3 $$GP = \frac{{{I_{blue}} - {I_{green}}}}{{{I_{blue}} + {I_{green}}}},$$

where *I*_*blue*_ and *I*_*green*_ correspond to the emission intensities at approximately 440 and 490 nm, respectively. If Laurdan is inside a membrane, its spectrum will move according to the bilayer's water content. Water content correlates with lipid packing and membrane fluidity^47–50^. For the GP value calculation, two-photon excitation is needed to minimize the severe photobleaching of the dye by one-photon excitation. This technique has been used before to measure membrane fluidity^46,47,51^*.*

#### Sample preparation

Human RBCs were obtained from the same healthy donor volunteer and stored at 4 °C. RBCs were centrifuged at 4000 rpm (1800 g), rinsed, and suspended to the original hematocrit (40% vol/vol) in PBS-saline buffer^48,51^. RBCs (at final hematocrit of 5% vol/vol) were incubated for 24 h at 37 °C in d-glucose at three concentrations (5.5, 12.5, and 25 mM). After incubation, RBCs were centrifuged at 4000 rpm (1800 g), and the pellet was rinsed three times with PBS for the fluorescence imaging measurements. d-glucose-treated erythrocytes were diluted with PBS to hematocrit 0.2% vol/vol and incubated with 1 µM Laurdan for 1 h at 37 °C. Samples were deposited in a microscope dish coated with poly-lysine (MatTek Co. Ashland, MA, USA) to allow the adhesion of the erythrocytes to the dish. Measurements under the microscope were obtained at room temperature.

#### Data acquisition

Intensity images were acquired in a two-photon microscope Zeiss LSM780 NLO using a 63X oil immersion objective (Plan-Apochromat). A Ti: Sapphire laser (Coherent, Chameleon) with 80 MHz repetition rate and tuned at 780 nm was used to excite the sample^48,51^, and the emission signal was received in two channels (412–464 nmand482–534 nm). The image size was 134.82 µm^2^ of 1024 × 1024 pixels.

### GP Data analysis

#### Determination of RBCs size

The size of the RBCs was determined with the intensity images from the blue channel (412–464 nm), and ImageJ software was used. A total of 112 cells were analyzed.

#### Determination of membrane fluidity

GP images were obtained by applying Eq. (3) to the intensity images and using ImageJ software. Twenty-five sets of images were obtained for each treatment and the control. From the GP image, the pixels attributed to the plasma membrane (5 pixels from the outer cellular limit) were selected, and the pixels histogram distribution ^46^ was obtained and analyzed ^45,46^. Average GP analysis gives the average membrane fluidity. The pixel histogram is fitted to a Gaussian distribution, and the center is reported as the average GP value for the membrane. Ten images are processed in this manner.

## Supplementary Information

Supplementary Information.
