# Supplementary material for: Correlated flickering of erythrocytes membrane observed with dual time resolved membrane fluctuation spectroscopy under different d-glucose concentrations
Source: Sci Rep. 2021 Jan 28;11:2429. doi: 10.1038/s41598-021-82018-5 (PMC7844050; doi:10.1038/s41598-021-82018-5)
Supplement: Supplementary file 1 — Supplementary Information. [file 41598_2021_82018_MOESM1_ESM.docx]

Supplementary Information

**Correlated flickering of erythrocytes membrane observed with dual time resolved membrane fluctuation spectroscopy under different D-glucose concentrations.**

J. Tapia^1^, N. Vera^1^, Joao Aguilar^3^, M. González^2^, S. A. Sánchez^3^, P. Coelho^4^, C. Saavedra^1^, J. Staforelli^1*🖂^

1 Departamento de Física, Universidad de Concepción, 4070386, 160-C, Concepción, Chile

2 Laboratorio de Investigación Materno-Fetal (LIMaF), Departamento de Obstetricia y Ginecología, Facultad de Medicina, Universidad de Concepción, Concepción, Chile.

3 Departamento de Polímeros, Facultad de Ciencias Químicas. Universidad de Concepción, Chile

4 Facultad de Ingeniería y Tecnología, Universidad San Sebastián, Lientur 1457, 4080871, Concepción, Chile.

Correspondence should be addressed to J. Staforelli^🖂^

🖂e-mail: [jstaforelli@udec.cl](mailto:jstaforelli@udec.cl)

**Supplementary Table 1.** Statistical P values obtained for the ANOVA tests in the analysis of the signal correlations.

| **Time Window** |  | **P-Value** |  |
| --- | --- | --- | --- |
|  | **5.5 vs. 12.5 mM** | **5 vs. 25 mM** | **12.5 vs. 25 mM** |
| 0.01 | 0.0316 | <0.0001 | 0.0425 |
| 0.1 | <0.0001 | <0.0001 | 0.9929 |
| $1$ | <0.0001 | <0.0001 | 0.9773 |
| 10 | <0.0001 | 0.0009 | 0.7727 |

**Supplementary Table 2.** Statistical P values obtained for the ANOVA tests in the analysis of the mechanical parameters obtained for the total RBC sample.

| **Signal** | **P value for** | **5.5 vs. 12.5 mM** | **5 vs. 25 mM** | **12.5 vs. 25 mM** |
| --- | --- | --- | --- | --- |
|  | Amplitude | <0.0001 | <0.0001 | 0.9692 |
| $S_{\vert V\rangle}$ | κ | 0.0099 | 0.0017 | 0.7591 |
|  | σ | 0.0006 | 0.0059 | 0.9418 |
|  | Amplitude | 0.0035 | 0.0582 | 0.7978 |
| $S_{\vert H\rangle}$ | κ | 0.6476 | 0.0912 | 0.4719 |
|  | σ | 0.0633 | 0.5433 | 0.6236 |

**Discussion about the consequences of noises in d-TRMFS measurements:**

To analyze the consequences of the noise on the signals measures in d-TRMFS, we must focus on what happens in the sample plane with the measurement signals. For this, consider two sources of noise. The first one is composed mainly of mechanical noises coming from external sources, such as sound from equipment or foot traffic in the building. The second source corresponds to the laser drift. Now, we will analyze and discuss the consequences of both noise sources on the measurements and correlations.

The red blood cell membrane rim can be thought of as a phase object that scatters a fraction of each measuring beam causing an interference pattern in the OL2 back focal plane (Fig. 1A). If mechanical perturbations are disturbing the RBC, we expect that its whole structure moves to the left or right, which would cause a displacement of both interference patterns in the same direction. To show this situation, consider Fig S.1A, where an RBC is being measured in the d-TRMFS setup. If RBC is disturbed for mechanical effects, its position could move to the left or right, as shown in figure S.1 B and S.1C. The behavior of the measurement signals $S_{|H\rangle}$ and $S_{|V\rangle}$ are shown in figures S.1D and S.1E for both cases considering the calibration curves in Fig. 1E and 1F.

Considering figure S.1B, if the RBC moves to the left, $S_{|H\rangle}$ would be further inside the membrane, and $S_{|V\rangle}$ would tend to come out of the membrane. In this case, the QPD signal values of the $S_{|H\rangle}$ and $S_{|V\rangle}$ would increase, as shown in figure S.1D. On the other hand, considering figure S.1C, if the RBC moves to the right, $S_{|H\rangle}$ would be further outside of the membrane, and $S_{|V\rangle}$ would tend to come in. Thus, the QPD signal values of the $S_{|H\rangle}$ and $S_{|V\rangle}$ would decrease, as shown in figure S.1E. Both for the coordinated displacement to the left or right, the signals would have a positive correlation.

For the laser drift, a more detailed explanation is given, based on the number of reflections and its consequences on the reflection parity (1). In our setup, shown in Fig 1A (in the main manuscript), a laser beam is separated into two different portions and recombined again by an optical arrangement composed of a PBS2, PBS3, PZM1, and PZM2. The beam portion transmitted through PBS2, corresponding to $S_{|H\rangle}$, is reflected only once at PZM1, whereas the reflected beam, corresponding to $S_{|V\rangle}$, suffers three reflections. Thus, both beams had an odd number of reflections when coming out from PBS3, and therefore have the same parity. The next reflection on the optical path occurs at DF1, where both beams reflect at once, hence both have the same parity. When going through the sample, both beams are located at each side of the red blood cell, as shown in figure 1B in the sample plane close-up. If there is laser drift in the system, the beams move along the same direction over the sample plane owing to the parity. For this reason, the variation of the signal values is as in figure S.1D and S.1E, depending on the laser drift. Therefore, a positive correlation should be expected if there is a laser drift.


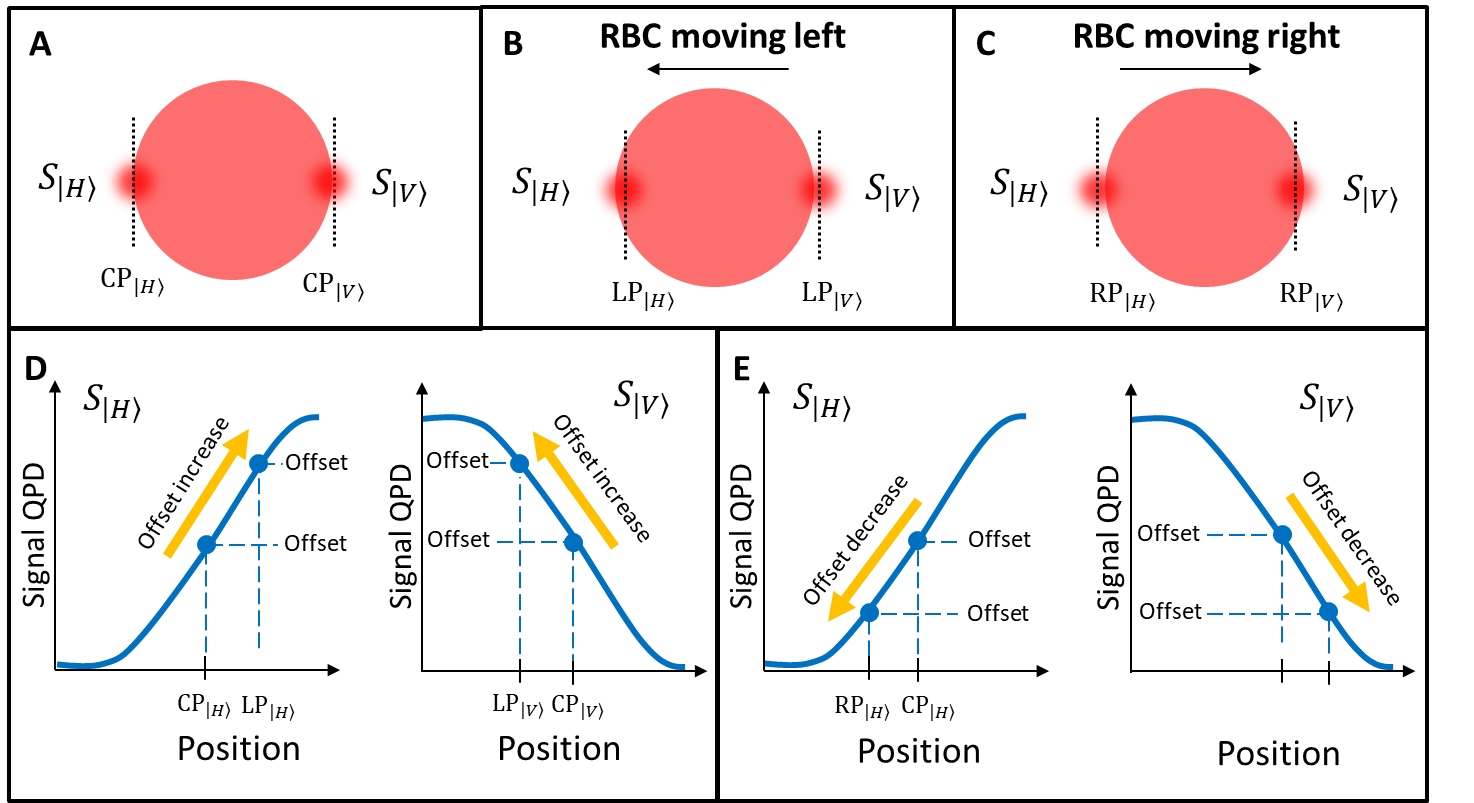


**Figure S.1**. Noise and its consequences in the signal values. **A** shows the centered beams ($CP$) in the membrane rim. **B** shows the beam centers when the cell is displaced to the left (LP). **C** are the points when the cell is displaced to the right (RP). **C** Displacement of the QPD signals from CP to LP. **D** Displacement for the QPD signals from CP to RP.

**References**

1. Greivenkamp, J. E.. *Field guide to geometrical optics. (*SPIE, 2004). 37-38
